# Characteristics of Multidrug Resistant *Shigella* and *Vibrio cholerae* O1 Infections in Patients Treated at an Urban and a Rural Hospital in Bangladesh

**DOI:** 10.1155/2013/213915

**Published:** 2013-12-22

**Authors:** Sumon Kumar Das, Erik H. Klontz, Ishrat J. Azmi, Abu I. M. S. Ud-Din, Mohammod Jobayer Chisti, Mokibul Hassan Afrad, Mohammad Abdul Malek, Shahnawaz Ahmed, Jui Das, Kaisar Ali Talukder, Mohammed Abdus Salam, Pradip Kumar Bardhan, Abu Syed Golam Faruque, Karl C. Klontz

**Affiliations:** ^1^Centre for Nutrition and Food Security (CNFS), International Centre for Diarrheal Disease Research, Bangladesh (icddr,b), 68 Shaheed Tajuddin Ahmed Sarani, Mohakhali, Dhaka 1212, Bangladesh; ^2^Office of Analytics and Outreach, Center for Food Safety and Applied Nutrition, Food and Drug Administration, 5100 Paint Branch Parkway, College Park, MD 20740, USA

## Abstract

We determined the frequency of multidrug resistant (MDR) infections with *Shigella* spp. and *Vibrio cholerae* O1 at an urban (Dhaka) and rural (Matlab) hospital in Bangladesh. We also compared sociodemographic and clinical features of patients with MDR infections to those with antibiotic-susceptible infections at both sites. Analyses were conducted using surveillance data from the International Centre for Diarrhoeal Disease Research, Bangladesh (icddr,b), for the years 2000–2012. Compared to patients with antibiotic-susceptible for *Shigella* infections, those in Dhaka with MDR shigellosis were more likely to experience diarrhea for >24 hours, while, in Matlab, they were more likely to stay inhospital >24 hours. For MDR shigellosis, Dhaka patients were more likely than those in Matlab to have dehydration, stool frequency >10/day, and diarrheal duration >24 hours. Patients with MDR *Vibrio cholerae* O1 infections in Dhaka were more likely than those in Matlab to experience dehydration and stool frequency >10/day. Thus, patients with MDR shigellosis and *Vibrio cholerae* O1 infection exhibited features suggesting more severe illness than those with antibiotic-susceptible infections. Moreover, Dhaka patients with MDR shigellosis and *Vibrio cholerae* O1 infections exhibited features indicating more severe illness than patients in Matlab.

## 1. Introduction

*Shigella* and *Vibrio cholerae* O1 are widely recognized causes of dysentery and acute watery diarrhea, respectively [1, 2]. Both have been responsible for producing epidemics [3] and often require antibiotic treatment to mitigate the severity of disease [4–6]. For *Shigella* infections, in particular, increasing antibiotic resistance has led to fewer antibiotics capable of producing bacteriostatic or bactericidal minimum inhibitory concentrations (MICs) [5, 7]. The challenge is compounded by the fact that while the regional prevalence of infection may be similar, rates of antibiotic resistance may differ substantially from one nation to another [5, 8].

Since 1979 and 2000, the International Centre for Diarrhoeal Disease Research, Bangladesh (icddr,b), has tested a systematic random sample of patients seeking care at an urban Dhaka Hospital and rural Matlab Treatment Centre for a spectrum of diarrhea-causing pathogens, including *Shigella* and *Vibrio cholerae* O1 [9]. Antimicrobial susceptibility patterns for these two pathogens are determined to inform clinicians about appropriate antibiotic treatment options [10]. Underscoring the need for antimicrobial sensitivity testing is the emergence of multidrug resistance (MDR), defined here as isolates resistant to ≥3 drugs [11–13]. Infections due to MDR strains are important not only because they are more difficult to treat but also because they may lead to higher fatality rates [14, 15]. Resource constraints in developing nations such as Bangladesh have limited the amount of information about the clinical features of MDR *Shigella* and *Vibrio cholerae* O1 infections. To address this gap, we determined the proportion of patients exhibiting MDR *Shigella* or *Vibrio cholerae* O1 infections at icddr,b from 2000 to 2012. We also studied sociodemographic and clinical features of MDR infections compared to antibiotic-susceptible infections, and compared these features in patients with MDR infections treated at the urban (Dhaka) and rural (Matlab) hospitals.

## 2. Materials and Methods

### 2.1. Study Sites

#### 2.1.1. Dhaka Hospital

Dhaka Hospital is located in the capital of Bangladesh. The hospital was established in 1962 by icddr,b, and currently provides free care and treatment to around 140,000 patients each year. The Diarrheal Disease Surveillance System (DDSS), approved by the Research Review Committee and Ethical Review Committee, has operated at icddr,b since 1979 to collect data on patient populations. From 1979 to 1995, microbiologic tests for a spectrum of diarrheal etiologies were conducted on a systematic sample of 4% of patients who attended icddr,b, whereas, since 1996, 2% of patients have been sampled to account for a near-doubling in the number of patients seeking care at icddr,b. A structured questionnaire is used to collect information on clinical, epidemiological, and demographic characteristics of patients, the feeding practices of infants and young children, and the use of drug and fluid therapy at home.

#### 2.1.2. Matlab Hospital

Since 1963, icddr,b has maintained a facility in rural Matlab, located about 55 km from Dhaka, for treating patients with diarrhea in the region. Each year, the facility provides free treatment to 20,000 patients with diarrhea. At Matlab, unlike at the Dhaka facility, every patient with diarrhea is screened for the spectrum of diarrheal pathogens assessed as part of the DDSS.

### 2.2. Study Sample

**Figure 1:**
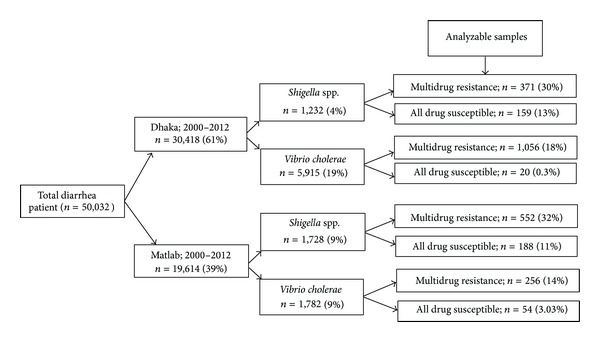
Sampling frame, testing of patients with diarrheal disease for multidrug resistant infections due to *Shigella* spp. or *Vibrio cholerae* O1, International Centre for Diarrhoeal Disease Research (icddr,b), Bangladesh, 2000–2012.

Clinical and epidemiologic details were abstracted from the electronic data archive of DDSS for patients with diarrhea treated at icddr,b facilities in Dhaka and Matlab from 2000 to 2012. We compared sociodemographic and clinical features of patients from whom MDR *Shigella* spp. or *Vibrio cholerae* O1 was recovered with patients whose diarrheal stools yielded susceptible strains of the respective pathogens.  Figure 1 illustrates the sampling frame for the study.

### 2.3. Laboratory Methodology

Fresh whole stool specimens collected from patients were examined at either the central icddr,b laboratory in Dhaka or at the Matlab clinical laboratory. Using standard laboratory methods described elsewhere [16, 17], each specimen was screened for common enteric pathogens, including *Shigella* spp. and *Vibrio cholerae *O1.

Bacterial susceptibility to antimicrobial agents was determined by the disk diffusion method as recommended by the Clinical Laboratory Standards Institute (CLSI 2010, June update) with commercial antimicrobial discs (Oxoid, Basingstoke, UK) [18]. The antibiotic discs used in this study for *Shigella* spp. included ampicillin (10 *μ*g), mecillinam (25 *μ*g), nalidixic acid (30 *μ*g), trimethoprim-sulfamethoxazole, (25 *μ*g), and ciprofloxacin (5 *μ*g); and for *V. cholerae *O1: tetracycline (30 *μ*g), trimethoprim-sulfamethoxazole (25 *μ*g), erythromycin (15 *μ*g), ciprofloxacin (5 *μ*g), and azithromycin (15 *μ*g) (Dhaka only) [18].

### 2.4. Data Analysis

Data were analyzed using Statistical Package for Social Sciences (SPSS) Windows (Version 15.2; Chicago, IL) and Epi Info (Version 6.0, USD, Stone Mountain, GA). We compared differences in proportions using the Chi-square test. A probability value (*P* value) of <0.05 was considered to confer statistical significance. Magnitudes of association were determined by estimating odds ratios (OR) and 95% confidence intervals (CI). To identify MDR strains, we determined the frequency with which isolates exhibited resistance to ≥3 antibiotics. We first compared sociodemographic features of patients with MDR infections versus those with susceptible infections. Next, focusing solely on MDR infections, we determined whether patients treated in Dhaka differed from those in Matlab in terms of sociodemographic or clinical features. All statistically significant differences ascertained by univariate analysis were entered into a logistic regression model to calculate adjusted odds ratios, *P* values, and 95% confidence intervals.

## 3. Results

### 3.1. *Shigella* spp

From 2000 to 2012, the number of patients who yielded MDR *Shigella *spp. in Dhaka and Matlab was 371/1,232 (30%) and 552/1,728 (32%), respectively (Figure 1). *S. flexneri* was recovered from a greater proportion of patients in Matlab compared to Dhaka, whereas *S. boydii* was recovered from a greater proportion of patients in Dhaka versus Matlab (Table 1). Resistance to ampicillin + nalidixic acid + trimethoprim-sulfamethoxazole was the most common resistance pattern for patients treated at both Dhaka and Matlab (Table 2). In Dhaka, the percentages of isolates resistant to individual antibiotics were trimethoprim-sulfamethoxazole (94%), ampicillin (85%), nalidixic acid (91%), mecillinam (25%), and ciprofloxacin (31%). In Matlab, the percentages of isolates resistant to individual antibiotics were nalidixic acid (96%), trimethoprim-sulfamethoxazole (92%), ampicillin (87%), ciprofloxacin (32%), and mecillinam (12%).

In Dhaka, compared to patients with antibiotic-susceptible *Shigella* infections, those with MDR infections were more likely be male and to experience diarrhea for >24 hours (Table 3). In Matlab, patients with MDR *Shigella* infections were more likely than those with susceptible infections to stay in hospital >24 hours and to report having used antimicrobials at home prior to coming to hospital (Table 4). For MDR *Shigella* infections, patients in Dhaka were more likely than those in Matlab to be male and to have dehydration, frequency of stools >10/day, and duration of diarrhea >24 hours (Table 5).

### 3.2. *Vibrio cholerae* O1

From 2000 to 2012, the number of patients who yielded MDR *Vibrio cholerae *O1 in Dhaka and Matlab was 1,056/5,915 (18%) and 256/1,782 (15%), respectively (Figure 1). In Dhaka, resistance to trimethoprim-sulfamethoxazole + tetracycline + erythromycin + furazolidone was the most common resistance pattern, while in Matlab it was trimethoprim-sulfamethoxazole + tetracycline + erythromycin (Table 2). In Dhaka, the proportions of isolates resistant to individual antibiotics were trimethoprim-sulfamethoxazole (99%), furazolidone (99%), erythromycin (85%), tetracycline (98%), ciprofloxacin (5%), and azithromycin (5%). In Matlab, the percentages of isolates resistant to individual antibiotics were trimethoprim-sulfamethoxazole (99%), furazolidone (42%), erythromycin (65%), tetracycline (93%), and ciprofloxacin (<1%).

Patients with MDR *Vibrio cholerae* O1 infections in Dhaka were more likely than those in Matlab to experience a frequency of stools of >10/day, dehydration, and the presence in stools of 1–10 macrophages per high power field (Table 6).

## 4. Discussion

To our knowledge, few studies have assessed differences in sociodemographic and clinical features of multidrug resistant versus susceptible infections due to *Shigella* spp. within a single nation or to compare these features for multidrug resistant infections due to *Shigella* spp. and *Vibrio cholerae* O1 at an urban versus a rural treatment center [19]. Although multidrug resistant infections are a problem globally, the ease of availability of antibiotics to the public at large in nations such as Bangladesh may expedite the rate at which resistance develops [20]. In the present study, the proportion of multidrug resistant *Shigella* and *Vibrio cholerae* O1 isolates recovered from patients in urban Dhaka was similar to that in rural Matlab. Of concern, in both hospital settings, there was evidence of resistance to mecillinam and ciprofloxacin, drugs commonly used for the treatment of shigellosis and, in the case of ciprofloxacin, cholera. In general, the emergence of resistance poses a number of challenges by leading, in some instances, to an increase in morbidity and mortality and longer hospital stays, as a result of inadequate initial therapy or increased virulence [15]. Moreover, antibiotic resistance reduces choices for therapy and can cause health care costs to rise due to the need to use antimicrobial agents that are more expensive than those in current use.

While antibiotic resistance in both *Shigella* spp. and *Vibrio cholerae* O1 has serious ramifications, for *Shigella* spp. the phenomenon carries added weight given that susceptibility rarely returns after resistant strains have become endemic in a region [12, 21]. Although *S. dysenteriae* 1 frequently develops resistance to new antibiotics initially, resistance is often acquired subsequently in the other *Shigella* species. In contrast, *Vibrio cholerae* O1 strains often revert to antibiotic susceptibility [12]. This phenomenon was observed during the study period when, abruptly in late 2004, *Vibrio cholerae* O1 isolates at both Matlab and Dhaka demonstrated tetracycline resistance; however, two years later, in 2006, tetracycline susceptibility reappeared in large part [22].

We observed evidence that multidrug resistant infections due to *Shigella* spp. were associated with more severe illness compared to non-MDR strains. For example, patients in Dhaka from whom multiresistant *Shigella* spp. were recovered significantly were more likely to experience diarrhea for >24 hours compared to patients from whom susceptible isolates were recovered. Similarly, in Matlab, patients from whom multidrug resistant *Shigella* spp. was recovered are more likely than those with susceptible infections to stay in hospital >24 hours and to report having used antimicrobials at home prior to coming to hospital. We also found evidence of differing degrees of severity of multidrug resistant infections due to *Shigella* spp. and *Vibrio cholerae* O1 infections depending on whether patients resided in urban or rural areas of the country. For example, for infections with MDR *Shigella* spp., patients in Dhaka were more likely than those in Matlab to experience dehydration, a frequency of stools >10/day, and duration of diarrhea >24 hours. Similarly, patients with MDR *Vibrio cholerae* O1 infections in Dhaka were more likely than those in Matlab to experience dehydration, frequency of stools of >10/day, and the presence of macrophages in stools. Further studies are needed to corroborate whether patients with multidrug resistant infections due to *Shigella* spp. and *Vibrio cholerae* O1 who reside in urban areas are, in fact, at elevated risk of experiencing more severe illness than patients in rural areas, and, if the findings are borne out, what factors contribute to this phenomenon. In the meantime, we hypothesize that differences in sociodemographic, nutritional, and economic characteristics of urban versus rural populations may, in part, explain our observations, as may differences in sources of drinking water and water-sanitation practices.

The present study has several limitations. Hospital-based data of the type incorporated in the Diarrheal Disease Surveillance System may not adequately represent the ill population at large. For example, a segment of the population in Bangladesh, particularly in rural regions, is known to use medicinal plants and traditional healers as a first-line of health care to cure gastrointestinal disorders [23]. Consequently, it is possible that sociodemographic and clinical features of patients with shigellosis and cholera described here may not be completely representative of all infections that occurred in the study area and study period. In addition, the results of antibiotic resistance testing we used were largely qualitative—presence, absence—as opposed to a quantitative nature as provided by minimum inhibitory concentrations. Thus, we were unable to ascertain pathogen-antibiotic relationships, wherein resistance levels may have approached limits known to confer resistance. Finally, our results did not incorporate the role of virulence factors or genetic typing that could have added valuable insight into outcomes of interest.

Notwithstanding these limitations, we believe that our results justify further research to determine, first, whether multidrug resistant strains of *Shigella* and *Vibrio cholerae* O1 are associated with more severe infections than antibiotic-susceptible strains, and, if so, what factors increase the severity of infections, and second, whether multidrug resistant infections caused by these two pathogens produce more severe infections in urban versus rural regions. Answers to these questions may help in efforts to prevent infections due to* Shigella* spp. and *Vibrio cholerae* O1, both of which are often leading bacterial causes of diarrhea in developing nations.

## Figures and Tables

**Table 1:** Multidrug resistant (MDR) *Shigella* species recovered from patients in Dhaka and Matlab, International Centre for Diarrhoeal Disease Research (icddr,b), Bangladesh, 2000–2012.

*Shigella* spp.	Dhaka; *n* = 371 (%)	Matlab, *n* = 552 (%)	OR (95% CI) *P* value
*Shigella flexneri *	257 (69)	495 (90)	0.26 (0.18, 0.37) <0.001
*Shigella boydii *	68 (18)	33 (6)	0.28 (0.18, 0.45) <0.001
*Shigella sonnei *	22 (6)	17 (3)	1.98 (0.99, 3.97) 0.051
*Shigella dysenteriae* 1	4 (1)	1 (0.2)	6.01 (0.63, 141.61) 0.016
*Shigella dysenteriae *	20 (5)	6 (1)	5.19 (1.95, 14.56) <0.001

**Table 2:** Multidrug resistant *Shigella* spp. and *Vibrio cholerae* O1 recovered from patients with diarrhea, Dhaka and Matlab, International Centre for Diarrhoeal Research Bangladesh (icddr,b), 2000–2012.

Antimicrobials	*Shigella *spp.			*Vibrio cholerae *		
	Dhaka; *n* = 371 (%)	Matlab; *n* = 552 (%)	OR (95% CI) *P*	Dhaka; *n* = 1056 (%)	Matlab; *n* = 256 (%)	OR (95% CI) *P*
AMP + NAL + TMST	203 (55)	301 (56)	0.94 (0.72, 1.24) 0.715	—	—	—
AMP + NAL + MEC	4 (1)	19 (3)	0.31 (0.09, 0.96) 0.040	—	—	—
AMP + NAL + CIP	1 (0.3)	20 (4)	0.07 (0.00, 0.51) <0.001	—	—	—
AMP + TMST + MEC	—	21 (4)	—	—	—	—
AMP + TMST + CIP	18 (5)	1 (0.2)	28.10 (3.96, 567.52) <0.001	—	—	—
AMP + CIP + MEC	24 (7)	—	—	—	—	—
AMP + TMST + AZI	5 (1)	—	—	—	—	—
AMP + CIP + AZI	2 (0.5)	—	—	—	—	—
AMP + MEC + AZI	2 (0.5)	—	—	—	—	—
TMST + CIP + AZI	3 (1)	—	—	—	—	—
TMST + MEC + AZI	2 (0.3)	—	—	—	—	—
CIP + MEC + AZI	1 (0.3)	—	—	—	—	—
NAL + CIP + MEC	—	20 (4)	—	—	—	—
NAL + TMST + MEC	4 (1)	1 (0.2)	6.01 (0.63, 141.61) 0.163	—	—	—
NAL + TMST + CIP	22 (6)	67 (12)	0.48 (0.28, 0.82) 0.005	—	—	—
TMST + CIP + MEC	3 (1)	3 (1)	1.49 (0.24, 9.27) 0.689	—	—	—
TET + TMST + FUR	—	—	—	103 (10)	89 (35)	0.20 (0.14, 0.29) <0.001
TET + FUR + ERY	—	—	—	—	1 (0.4)	—
TMST + FUR + ERY	—	—	—	7 (1)	17 (7)	0.09 (0.03, 0.24) <0.001
TMST + FUR + CIP	—	—	—	—	—	—
TET + TMST + ERY	—	—	—	38 (4)	148 (58)	0.03 (0.02, 0.04) <0.001
AMP + TMST + CIP + AZI	7 (2)	—	—	—	—	—
AMP + TMST + MEC + AZI	2 (1)	—	—	—	—	—
AMP + CIP + MEC + AZI	1 (0.3)	—	—	—	—	—
AMP + CIP + NAL + AZI	1 (0.3)	—	—	—	—	—
AMP + NAL + TMST + CIP	19 (5)	67 (12)	0.39 (0.22, 0.68) <0.001	—	—	—
AMP + NAL + TMST + MEC	3 (1)	—	—	—	—	—
AMP + NAL + CIP + MEC	3 (1)	4 (1)	1.12 (0.20, 5.92) 1.000	—	—	—
AMP + CIP + TMST + MEC	9 (3)	—	—	—	—	—
TMST + NAL + CIP + MEC	1 (0.3)	3 (1)	0.49 (0.02, 5.32) 0.652	—	—	—
TMST + TET + ERY + FUR	—	—	—	906 (86)	—	—
TMST + ERY + CIP + FUR	—	—	—	1 (0.1)	—	—
TMST + TET + ERY + AZI	—	—	—	1 (0.1)	—	—
AMP + TMST + NAL + CIP + MEC	24 (7)	16 (3)	2.32 (1.16, 4.64) 0.014	—	—	—
AMP + TMST + CIP + MEC + AZI	7 (1)	—	—	—	—	—
FUR + ERY + CIP + TMST + TET	—	—	—	—	1 (0.4)	—

AMP: Ampicillin; AZI: azithromycin; CIP: ciprofloxacin; ERY: erythromycin; FUR: furazolidin; MEC: mecillinam; NAL: nalidixic acid; TET: tetracycline; TMST: trimethoprim-sulfamethoxazole.

**Table 3:** Sociodemographic and clinical factors among patients with shigellosis, by drug resistance status, Dhaka, International Centre for Diarrhoeal Disease Research, Bangladesh (icddr,b), 2000–2012.

Indicators	Multidrug resistant *n* = 371 (%)	Susceptible *n* = 159 (%)	OR (95% CI)	aOR (95% CI)
Male sex	225 (61)	111 (70)	6.72 (3.25, 14.23)*	1.58 (1.02, 2.43)*
Monthly family income ≥100 USD	345 (93)	147 (93)	1.08 (0.50, 2.31)	—
Slum residence	33 (9)	19 (12)	0.72 (0.38, 1.37)	—
Nonsanitary latrine	133 (36)	61 (38)	0.90 (0.60, 1.34)	—
Not treated water	249 (67)	103 (65)	1.11 (0.74, 1.67)	—
Vomiting	243 (66)	120 (76)	0.62 (0.40, 0.96)*	0.84 (0.52, 1.35)
Abdominal pain	206 (56)	76 (48)	1.36 (0.92, 2.01)	—
Fever (≥38°C)	40 (11)	7 (4)	2.62 (1.10, 6.57)*	2.18 (0.93, 5.12)
Bloody or mucoid stool	165 (45)	63 (40)	1.22 (0.82, 1.81)	—
Frequency of stool (>10/day)	189 (51)	75 (47)	1.16 (0.79, 1.72)	—
Duration of diarrhea (>24 hours)	262 (71)	87 (55)	1.99 (1.33, 2.97)*	1.73 (1.11, 2.69)*
Duration of stay in hospital >24 hrs	134 (37)	54 (35)	1.09 (0.72, 1.64)	—
Some or severe dehydration	211 (57)	84 (53)	1.18 (0.80, 1.74)	—
Use of intravenous saline for rehydration	77 (21)	27 (17)	1.28 (0.77, 2.14)	—
Use of antimicrobials at home	251 (68)	91 (57)	1.56 (1.05, 2.33)*	1.20 (0.78, 1.86)
Red blood cell (1 to >50)	241 (69)	76 (52)	2.07 (1.37, 3.13)*	0.77 (0.33, 1.83)
Faecal leukocyte (11 to >50)	272 (78)	88 (60)	2.34 (1.51, 3.62)*	1.62 (0.81, 3.24)
Macrophage (1 to 10)	219 (63)	62 (42)	2.29 (1.52, 3.46)*	0.92, 4.08)

*P < 0.05.

aOR: adjusted odds ratio.

**Table 4:** Sociodemographic and clinical factors among patients with shigellosis, by drug resistance status, Matlab, International Centre for Diarrhoeal Disease Research, Bangladesh (icddr,b), 2000–2012.

Indicators	Multidrug resistant *n* = 552 (%)	Susceptible *n* = 188 (%)	OR (95% CI)	aOR (95% CI)
Male sex	297 (54)	95 (51)	1.14 (0.81, 1.61)	—
Monthly family income ≥100 USD	257 (47)	79 (42)	1.20 (0.85, 1.70)	—
Nonsanitary latrine	486 (88)	165 (88)	1.03 (0.60, 1.75)	—
Not treated water	544 (99)	186 (99)	0.73 (0.11, 3.75)	—
Vomiting	273 (50)	99 (53)	0.88 (0.62, 1.24)	—
Abdominal pain	370 (67)	130 (69)	0.91 (0.62, 1.32)	—
Fever (≥38°C)	132 (24)	51 (27)	0.84 (0.57, 1.25)	—
Bloody or mucoid stool	425 (77)	126 (67)	1.65 (1.13, 2.40)*	1.23 (0.80, 1.87)
Frequency of stool (>10/day)	230 (42)	77 (41)	1.03 (0.73, 1.46)	—
Duration of diarrhea (>24 hours)	373 (68)	107 (57)	1.58 (1.11, 2.25)*	1.35 (0.92, 1.99)
Duration of stay in hospital >24 hrs	216 (40)	57 (31)	1.51 (1.04, 2.18)*	1.79 (1.23, 2.62)*
Some or severe dehydration	108 (20)	53 (28)	0.62 (0.42, 0.92)*	0.69 (0.45, 1.06)
Use of intravenous saline for rehydration	24 (4)	9 (5)	0.90 (0.39, 2.14)	—
Use of antimicrobials at home	33 (60)	88 (47)	1.73 (1.22, 2.45)*	1.48 (1.03, 2.12)*
Red blood cell (1 to >50)	509 (93)	154 (82)	2.80 (1.65, 4.73)*	2.12 (0.85, 5.43)
Faecal leukocyte (11 to >50)	527 (96)	166 (89)	3.17 (1.62, 6.22)*	1.78 (0.66, 4.85)
Macrophage (1 to 10)	451 (82)	136 (73)	1.74 (1.16, 2.62)*	0.92 (0.51, 1.65)

*P < 0.05.

aOR: adjusted odds ratio.

**Table 5:** Sociodemographic and clinical features of patients with multidrug resistant *Shigella* infections in Dhaka compared to those in Matlab, International Centre for Diarrhoeal Disease Research, Bangladesh (icddr,b), 2000–2012.

Indicators	OR (95% CI)	aOR (95% CI)
Male sex	1.24 (0.94, 1.64)	1.52 (1.08, 2.16)*
Vomiting	1.94 (1.47, 2.57)*	1.26 (0.87, 1.82)
Abdominal pain	0.61 (0.46, 0.81)*	0.87 (0.59, 1.28)
Fever (≥38°C)	0.38 (0.26, 0.57)*	0.39 (0.25, 0.65)*
Bloody or mucoid stool	0.24 (0.18, 0.32)*	0.39 (0.26, 0.62)*
Frequency of stool (>10/day)	1.45 (1.11, 1.91)*	1.83 (1.28, 2.59)*
Duration of diarrhea (>24 hours)	1.15 (0.86, 1.55)	1.64 (1.07, 2.52)*
Duration of stay in hospital >24 hrs	0.90 (0.68, 1.19)	0.84 (0.58, 1.20)
Some or severe dehydration	5.42 (4.00, 7.36)*	5.61 (3.75, 8.39)*
Use of intravenous saline for rehydration	5.80 (3.51, 9.66)*	1.53 (0.79, 2.94)
Use of antimicrobials at home	1.38 (1.03, 1.83)*	1.42 (0.97, 2.08)
Red blood cell (1 to >50)	0.17 (0.11, 0.26)*	0.21 (0.08, 0.52)*
Faecal leukocyte (11 to >50)	0.14 (0.08, 0.24)*	0.42 (0.17, 1.07)
Macrophage (1 to 10)	0.36 (0.26, 0.50)*	1.81 (0.98, 3.35)

*P < 0.05.

aOR: adjusted odds ratio.

**Table 6:** Sociodemographic and clinical features of patients with multidrug resistant *Vibrio cholerae *O1 infections in Dhaka compared to those in Matlab, International Centre for Diarrhoeal Disease Research, Bangladesh (icddr,b), 2000–2012.

Indicators	Dhaka; *n* = 1,056 (%)	Matlab; *n* = 256 (%)	OR (95% CI)	aOR (95% CI)
Male sex	607 (56)	126 (46)	1.39 (1.05, 1.85)*	1.40 (0.79, 2.48)
Vomiting	976 (92)	216 (84)	2.26 (1.47, 3.46)*	1.86 (0.71, 4.84)
Abdominal pain	448 (42)	131 (51)	0.70 (0.53, 0.93)*	0.50 (0.28, 0.89)*
Fever (≥38°C)	7 (1)	16 (6)	0.10 (0.04, 0.26)*	0.09 (0.02, 0.57)*
Watery stool	1048 (99)	245 (96)	5.88 (2.17, 16.19)*	0.27 (0.00, 18.62)
Frequency of stool (>10/day)	592 (56)	237 (93)	2.31 (1.73, 3.10)*	3.32 (1.78, 6.17)*
Duration of diarrhea (>24 hours)	369 (35)	75 (29)	1.30 (0.95, 1.76)*	0.98 (0.49, 1.95)
Duration of stay in hospital >24 hrs	391 (39)	170 (67)	0.31 (0.23, 0.42)*	0.39 (0.22, 0.71)*
Some or severe dehydration	998 (94)	214 (84)	3.44 (2.20, 5.37)*	3.07 (1.13, 8.32)*
Use of intravenous saline for rehydration	828 (79)	152 (59)	2.58 (1.91, 3.48)*	1.90 (0.88, 4.11)
Use of antimicrobials at home	454 (43)	126 (49)	0.78 (0.59, 1.03)	1.35 (0.75, 2.45)
Red blood cell (1 to >50)	456 (44)	167 (66)	0.39 (0.29, 0.53)*	0.19 (0.09, 0.39)*
Faecal leukocyte (11 to >50)	508 (49)	198 (79)	0.26 (0.18, 0.36)*	0.35 (0.17, 0.73)*
Macrophage (1 to 10)	292 (28)	33 (13)	2.58 (1.72, 3.88)*	11.75 (4.87, 28.31)*

*P < 0.05.

aOR: adjusted odds ratio.
